# Projected Near-Future Levels of Temperature and *p*CO_2_ Reduce Coral Fertilization Success

**DOI:** 10.1371/journal.pone.0056468

**Published:** 2013-02-14

**Authors:** Rebecca Albright, Benjamin Mason

**Affiliations:** 1 Climate Change and Ocean Acidification, Australian Institute of Marine Science, Townsville, Queensland, Australia; Heriot-Watt University, United Kingdom

## Abstract

Increases in atmospheric carbon dioxide (*p*CO_2_) are projected to contribute to a 1.1–6.4°C rise in global average surface temperatures and a 0.14–0.35 reduction in the average pH of the global surface ocean by 2100. If realized, these changes are expected to have negative consequences for reef-building corals including increased frequency and severity of coral bleaching and reduced rates of calcification and reef accretion. Much less is known regarding the independent and combined effects of temperature and *p*CO_2_ on critical early life history processes such as fertilization. Here we show that increases in temperature (+3°C) and *p*CO_2_ (+400 µatm) projected for this century negatively impact fertilization success of a common Indo-Pacific coral species, *Acropora tenuis*. While maximum fertilization did not differ among treatments, the sperm concentration required to obtain 50% of maximum fertilization increased 6- to 8- fold with the addition of a single factor (temperature or CO_2_) and nearly 50- fold when both factors interact. Our results indicate that near-future changes in temperature and *p*CO_2_ narrow the range of sperm concentrations that are capable of yielding high fertilization success in *A. tenuis*. Increased sperm limitation, in conjunction with adult population decline, may have severe consequences for coral reproductive success. Impaired sexual reproduction will further challenge corals by inhibiting population recovery and adaptation potential.

## Introduction

Atmospheric carbon dioxide (*p*CO_2_) has increased from approximately 280 ppm to 390 ppm since the start of the industrial revolution due to anthropogenic activities such as the burning of fossil fuels, cement production, and land use changes [1]. Two major consequences of this increase have been a rise in global average temperature of 0.7°C, i.e., global warming, and a decrease in the average global surface ocean pH of 0.1 units (a 30% increase in acidity), i.e., ocean acidification or OA [1]. Projections, based on Special Report of Emissions Scenarios, suggest atmospheric CO_2_ concentration could reach up to 560 ppm by the year 2050 and over 800 ppm by 2100. This elevation in *p*CO_2_ will likely contribute to a further increase in global average surface warming of 1.1–6.4°C and a reduction in the average global surface ocean pH of between 0.14 and 0.35 units over the 21st century [1].

For coral reefs, the most likely consequences of ocean acidification and warming, respectively, are generally regarded as reduced calcification/reef accretion [2] and increased frequency and severity of coral bleaching (a breakdown of the symbiotic relationship between corals and their endosymbiotic algae) [3]. These concerns, in combination with recent trends in reef degradation caused by local stressors (e.g., overexploitation and pollution), have led to predicted future scenarios that range from spatially heterogenous declines dominated by shifts in community composition [4], [5] to global-scale loss of coral reefs [6], [7]. Recently there has been a growing awareness that rising ocean temperatures and declining pH may affect a variety of physiological and biological processes that span multiple life history stages [8]. Reproductive and early life history stages can be particularly vulnerable to environmental stress [9], and reduced reproduction and recruitment threaten reef resilience, i.e., the ability to recover post-disturbances. In light of the recent trends in reef loss, there is an urgent need to understand how global warming and ocean acidification will affect the reproductive capacity, and the recovery potential, of reef-building corals.

While many corals reproduce asexually, sexual reproduction is important for maintaining genetic diversity, populating denuded areas, determining the community structure of coral reefs, and replenishing reefs post disturbances. Corals have two primary strategies for sexual reproduction: (1) brooding, in which sperm are released into the water column and taken in by conspecifics for internal fertilization; and (2) broadcast spawning, in which eggs and sperm are released into the environment for external fertilization [10]. The majority of documented coral species are hermaphroditic broadcast spawners: they release gamete bundles, containing both eggs and sperm, into the water column and are reliant on external fertilization and larval development. Gamete bundles generally break apart approximately 30 minutes following release, whereupon gametes mix at the water's surface. Fertilized embryos develop into planula larvae over the course of hours to days before they are capable of settling on the reef [10].

As fertilization occurs externally, coral gametes are particularly vulnerable to the conditions of the surrounding water column. Consequently, fertilization success depends on a variety of factors, including sperm density, gamete age, water temperature, oxygen-availability, salinity, nutrients, pH, and *p*CO_2_ (reviewed in [11], [12]). As the oceans continue to warm and acidify, it becomes essential to understand the repercussions these changes will have on sexual reproduction. The number of studies investigating the independent effects of temperature and pH on early life history stages of corals is growing; however, few studies have investigated the interactive effects of these combined stressors [13].

Here we report the independent and combined effects of elevated temperature and *p*CO_2_ on the fertilization success of *Acropora tenuis* (Dana, 1846), a common species on upper reef slopes throughout the Indo-Pacific and Red Sea. Gametes were collected from naturally spawning colonies of *A. tenuis* and fertilized under control and experimental conditions. Two temperatures (27°C and 30°C) and two *p*CO_2_ levels (400 µatm and 800 µatm) were targeted to represent present-day conditions and a middle-of-the-road scenario projected for the end of the century [1]. The effects of these parameters were investigated across a range of sperm concentrations (10^2^–10^7^ sperm ml^−1^), spanning concentrations considered optimal for fertilization in broadcast-spawning corals, 10^5^–10^6^ sperm ml^−1^ [14].

## Methods

### Gamete collection and preparation

Gravid colonies of *A. tenuis* were collected from Orpheus Island, GBR (Permit G09/30237.1) and maintained in flow-through seawater in outdoor aquaria at the Australian Institute of Marine Science (AIMS), Townsville, Queensland. On evenings of predicted gamete release, colonies were placed in separate containment tanks and monitored for spawning activity. Spawning occurred on November 15, 2011 at approximately (1930 h). Upon release, gamete bundles were collected by pipette from each of five colonies and transferred to 50 ml falcon tubes for transport to the laboratory. Care was taken to avoid cross-contamination between genotypes. The volume of seawater in the tubes was adjusted to achieve 1∶4 (v/v) ratios of gametes∶seawater to ensure a concentrated stock solution (≥10^8^ sperm ml^−1^). Tubes were gently agitated to assist disintegration of gamete bundles, i.e., the release of eggs and sperm. Bundles disintegrated ∼60 min after release, whereupon eggs and sperm from each colony were separated.

#### Sperm handling

Concentrated sperm (∼10^8^ sperm ml^−1^) were removed from each tube by pipette and combined in a glass bottle to create a stock sperm solution consisting of equal parts sperm from each of the five parent colonies. The stock sperm solution was mixed by gentle inversion of the jar, and experimental sperm solutions were made by serial, ten-fold dilutions into 0.2 µm filtered treatment water (400 or 800 µatm, refer to ‘Seawater chemistry’ for details). Six dilutions (10^2^–10^7^ sperm ml^−1^) were made for each *p*CO_2_ level (400 and 800 µatm), totaling 12 sperm*CO_2_ solutions (10^2^–10^7^ sperm ml^−1^*400 µatm and 10^2^–10^7^ sperm ml^−1^*800 µatm); each dilution was gently inverted at least 5 times to ensure mixing. Borosilicate glass Schott bottles were used to maintain sperm viability and avoid abnormal cleavage of embryos that can occur with the use of plastics [14]. The sperm*CO_2_ solutions were then used to fill the replicate scintillation vials in each of the two temperature baths (see ‘Experimental design’ section). After, two 1-ml subsamples of each dilution in each sperm*CO_2_ combination were fixed in 10% formaldehyde for verification of sperm concentrations (ten replicate counts per subsample by hemocytometer).

#### Egg handling

Eggs from each parent colony were divided equally into new falcon tubes and washed 5–7 times with 0.2 µm filtered treatment water (400 or 800 µatm) to remove residual sperm. Once washed, equal volumes from each parent colony were combined to create two stock egg solutions (400 and 800 µatm) consisting of equal parts from each of the five parent colonies.

### Experimental design

**Figure 1:**
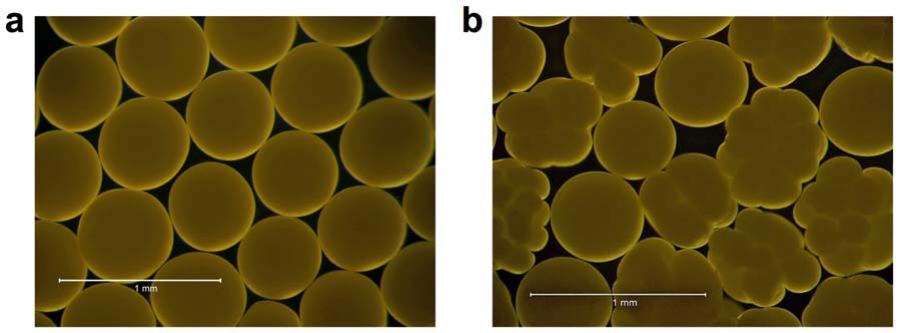
Representative images of unfertilized and fertilized *Acropora tenuis* embryos. Unfertilized eggs (a) are spherical and show no signs of cleavage while fertilized embryos (b) show distinct cell division.

A balanced factorial design was used to evaluate the effect of temperature and CO_2_ on the fertilization curve of *A. tenuis*. The design consisted of 144 scintillation vials: 2 CO_2_ levels (400 and 800 µatm)×2 temperatures (27 and 30°C)×6 sperm concentrations (10^2^, 10^3^, 10^4^, 10^5^, 10^6^, and 10^7^ sperm ml^−1^)×6 replicates (N = 6). Fifteen ml of each sperm*CO_2_ combination (see ‘Sperm handling’ section) was transferred (Pipetaid, Gilson) into each of 6 replicate 20-mL glass scintillation vials for each of the four CO_2_*T treatments. Twelve additional vials (three per CO_2_*T treatment) were dedicated for no-sperm controls and filled with 20-mL of sperm-free seawater. Scintillation vials were nested within re-circulating water baths for temperature control during the experiment (±0.2°C). Temperatures within the vials were allowed to equilibrate (∼30 min.) to the treatment levels prior to the addition of eggs. Thirty microliters of eggs (∼300 eggs) were added to each vial. To avoid sperm contamination (i.e., introduction of additional sperm between treatments), the addition of eggs was conducted in order from the lowest to highest sperm concentration, and tips were changed between sperm concentrations. Vials were gently swirled, and eggs were left to fertilize and develop. Fertilization experiments were initiated within 3 h of spawning. Embryos were sub-sampled at 3 hours (∼16–32 cell stage) and fixed in 10% formaldehyde; the samples were collected in the same order in which the eggs were introduced to ensure that gametes in all treatments were allowed equal sperm-egg contact time. Subsequently, 200–300 eggs/embryos from each subsample were examined using a dissecting microscope and scored as either fertilized (showing normal cleavage patterns of cell division) or unfertilized (showing no signs of cleavage) (Fig. 1).

### Model

**Figure 2:**
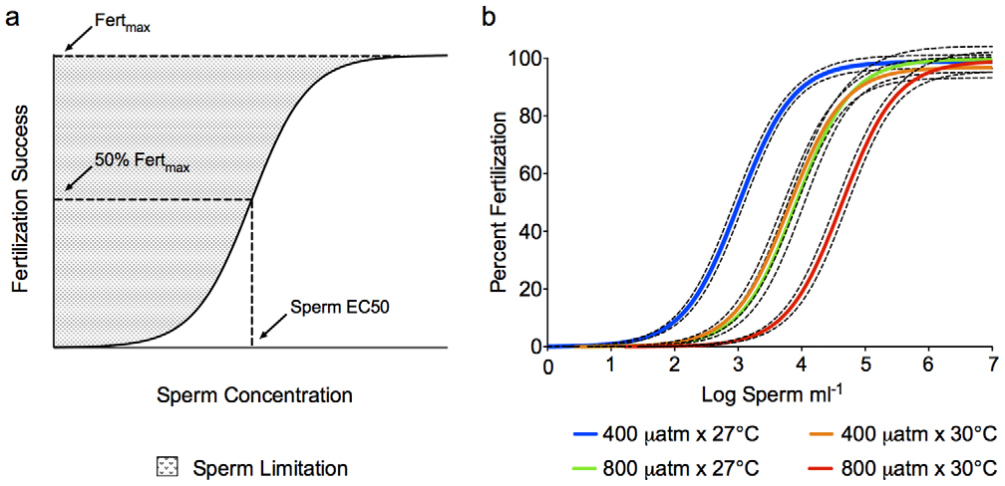
Theoretical and actual fertilization curves for *Acropora tenuis*. Schematic (a) showing the graphical representation of Fert_max_ and Sperm EC50, the sperm concentration at which 50% of Fert_max_ is achieved. The shading represents the region of sperm limitation resulting in less than maximal fertilization success (adapted from Marshall [15]). Fertilization curves (b) of *Acropora tenuis* by temperature and CO_2_ treatments. Dashed black lines indicate 95% confidence intervals for each curve. Parameter estimates are given in Table 3.

Fertilization curves in broadcast spawners are typically bell-shaped, in which sperm availability limits fertilization success at lower sperm concentrations, and polyspermy is thought to limit fertilization success at higher sperm concentrations [15]. In our study, fertilization success reached an asymptotic maximum, and we did not observe a decline in fertilization at higher sperm concentrations. Accordingly, sigmoidal dose-response curves were fit to the data, defined as follows: % Fert = % Fert_max_/(1+10^LogEC50-LogSC^), where % Fert is the percent fertilization at a given sperm concentration (SC), % Fert_max_ is the asymptotic average maximum percent fertilization, and EC50 is the ‘half maximal effective concentration’, or the sperm concentration that yields 50% of Fert_max_ (Fig. 2a). The model used follows the general equation for a standard, three parameter sigmoidal dose-response curve in which the bottom asymptote has been constrained to zero (0% Fert at 0 sperm ml^−1^) and the top asymptote, % Fert_max_, has been constrained to less than or equal to 100%. Nonlinear regressions were fit separately for each CO_2_*T treatment using least squares residuals. For each parameter, Fert_max_ and LogEC50, best-fit values were compared between treatments using One-way ANOVAs. Where significant differences were detected, Tukey's Multiple Comparisons tests were conducted to determine which treatments differed from one another (Table S1).

### Respiratory alterations of CO_2_ treatments

To test whether the addition of sperm to seawater caused respiratory alterations of desired CO_2_ levels, we measured pH in each T*CO_2_ combination for sperm concentrations 10^3^–10^7^ sperm ml^−1^ at the start and end of the experiment.

Changes were not measured in 10^2^ sperm ml^−1^ as preliminary work showed that changes in this concentration were comparable to seawater controls. So as not to disturb fertilization and/or embryonic development in the scintillation vials, separate 30 ml, screw-top glass vials, were used for pH measurements. Twenty milliliters of each sperm*CO_2_ combination were added to each vial (4 CO_2_*T treatments×5 sperm concentrations×2 replicates = 40 vials) and nested in the same water baths as the fertilization vials. Preliminary work indicated that chemical changes were highly consistent within a sperm concentration and that a sample size of 2 was sufficient to accurately document changes. pH was measured in each vial (SC*T*CO_2_ combination) using an Orion ROSS combination pH electrode (Thermo Scientific) calibrated at 27°C and 30°C against a seawater TRIS buffer [16] (SOP6a).

### Seawater chemistry

One ambient (400 µatm) and one elevated *p*CO_2_ concentration (800 µatm) were chosen for the study based on near-future projections determined by the Intergovernmental Panel on Climate Change (IPCC) [1]. These may represent conservative *p*CO_2_ levels, as modified projections (e.g., Representative Concentration Pathway (RCP) 8.5) used in the forthcoming Assessment Report (AR5) by the IPCC indicate that atmospheric CO_2_ may exceed 1,000 ppm by the end of this century [17].

Seawater chemistry was manipulated via direct bubbling with carbon dioxide-enriched air into two separate aquaria. The control was bubbled with outside air. Treatment water was filtered (0.2 µm) before use in experiments, to minimize changes of *p*CO_2_ due to background, microbial respiration. Duplicate water samples of each *p*CO_2_ treatment were collected prior to the start of the fertilization experiment and poisoned with mercuric chloride (0.05% by volume) to verify distinct treatments. Samples were analyzed for total alkalinity (TA) and dissolved inorganic carbon (DIC) using a VINDTA 3C® (Versatile INstrument for the Determination of Total dissolved inorganic carbon and Alkalinity, Marianda). Accuracy was checked against certified seawater reference material (from A. Dickson, Scripps Institute of Oceanography, Batch 106). *p*CO_2_, pH (total scale), and Ω_arag_ were computed from TA, DIC, temperature, and salinity using the CO2SYS program (from E. Lewis, Brookhaven National Laboratory); dissociation constants for carbonate and boric acid were determined as described in Albright et al. [18].

## Results

### Seawater chemistry

Actual chemical and physical conditions of the seawater used in fertilization assays are presented in Table 1. *p*CO_2_ levels differed slightly between temperature treatments due to the affect of temperature on the solubility of CO_2_.

**Table 1:** Physical and chemical conditions of seawater used to fill experimental vials for fertilization experiments (Mean ± 1 SD, N = 2)^a^.

Treatment	T* (°C)	Salinity*	TA* (µmol kg^−1^)	pH_T_	*p*CO_2_ (µatm)	HCO_3_^−^ (µmol kg^−1^)	CO_3_^−2^ (µmol kg^−1^)	CO_2_ (µmol kg^−1^)	TCO_2_* (µmol kg^−1^)	Ω_arag_
400 µatm×27°C (control)	27.0±0.2	36.0±0.2	2327±2	8.013±0.001	434±1	1787±2	219.5±0.1	11.63±0.02	2018±2	3.491±0.001
400 µatm×30°C	30.0±0.2	36.0±0.2	2327±2	7.970±0.001	488±1	1786±2	220.3±0.1	12.19±0.02	2018±2	3.559±0.001
800 µatm×27(C	27.0(0.2	36.0(0.2	2322(2	7.783(0.001	815(2	1972(2	142.5(0.1	21.83(0.03	2137(2	2.267(0.001
800((atm(30(C	30.0(0.2	36.0(0.2	2322(2	7.741(0.001	912(2	1970(2	143.6(0.1	22.77(0.03	2137(2	2.320(0.001

a Asterisks represent parameters that were directly measured; remaining parameters were calculated using CO2SYS (see ‘Methods’).

Error represents the analytical error of the TA and DIC analyses.

### Fertilization model

Percent fertilization by sperm concentration and treatment is presented in Table 2, and model parameters for nonlinear regressions are presented in Table 3. Maximum fertilization (Fert_max_) did not differ among treatments (F_3,148_ = 0.502, P = 0.681), but the fertilization curve shifted horizontally such that the sperm concentration required to achieve half of the maximum fertilization (EC50) increased with the addition of experimental factors (F_3,148_ = 141.7, P<0.0001) (Fig. 2b; Table 3). Fifty percent of Fert_max_ was attained at 1.0×10^3^ sperm ml^−1^ under control conditions (400 µatm and 27°C). Independent stressors, i.e., increasing temperature by 3°C or *p*CO_2_ by 400 µatm, resulted in a six- to eight-fold increase in EC50 (6.3×10^3^–8.2×10^3^ sperm ml^−1^), with no significant difference between the two factors (Table 3; Table S1). The combined effects resulted in a five- to seven- fold increase above the single factors, and a near 50-fold increase in EC50 relative to controls (4.3×10^4^ sperm ml^−1^) indicating a multiplicative interaction between temperature and CO_2_.

**Table 2:** Percent fertilization by sperm concentration and treatment (Mean ± SEM, N = 6).

	No Sperm Control	5.21×10^2^ sperm ml^−1^	5.51×10^3^ sperm ml^−1^	5.82×10^4^ sperm ml^−1^	6.15×10^5^ sperm ml^−1^	6.49×10^6^ sperm ml^−1^	6.86×10^7^ sperm ml^−1^
400 µatm×27°C	0.9±0.6	33.9±4	82.96±5	98.3±0.5	97.7±0.4	98.6±0.4	98.8±0.3
800 µatm×27°C	1.5±0.7	2.2±0.4	39.37±10	90.93±3	98.3±0.5	98.0±0.6	98.7±0.3
400 µatm×30°C	0.0±0.0	19.8±4	42.88±5	83.6±2	96.6±0.7	98.1±0.5	98.1±0.3
800 µatm×30°C	1.3±0.1	1.4±0.5	3.14±0.5	59.7±6	94.9±0.5	96.3±0.6	98.3±0.3

Self fertilization, ranging from 0.5% [44] to 2–3% [45], has been reported to occur in this species and is likely responsible for the low levels of fertilization in the no-sperm controls.

**Table 3:** Parameter estimates for nonlinear regressions of fertilization data^a^.

	400 µatm×27°C	800 µatm×27°C	400 µatm×30°C	800 µatm×30°C
Best-fit values				
EC50	1.0×10^3^	8.2×10^3^	6.3×10^3^	4.3×10^4^
%Fert_max_	99	100	97	99
95% CI				
EC50	8.3×10^2^–1.2×10^3^	5.9×10^3^–1.1×10^4^	4.8×10^3^–8.2×10^3^	3.4×10^4^–5.4×10^4^
%Fert_max_	96–100	95–100	93–100	96–100
Degrees freedom	37	37	37	37
R^2^	0.97	0.95	0.95	0.98

%Fert_max_ did not differ significantly between treatments. However, EC50 values significantly differed between all treatments except 400 µatm×30°C and 800 µatm×27°C. See Table S1 for results of Tukey's Multiple Comparisons.

a EC50 is the ‘half maximal effective concentration’, or the sperm concentration that yields 50% of the %Fert_max_; %Fert_max_ is the asymptotic average maximum percent fertilization.

### Respiratory alterations of CO_2_ treatments

**Figure 3:**
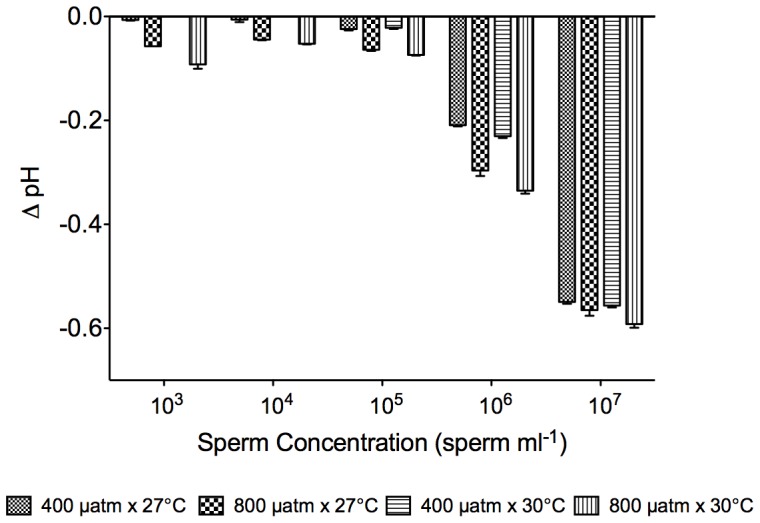
Respiratory alterations of target pH levels by sperm concentration and treatment (Mean ± 1 SD). Despite the rapid changes in pH at higher sperm concentrations (10^6^ and 10^7^ sperm ml^−1^), high levels of fertilization (≥95%) were achieved. See text for details.

We monitored pH at the beginning and end of our experiment to determine if respiratory activity by sperm altered our target *p*CO_2_/pH levels. The largest changes occurred in the ∼90 minutes immediately following the addition of sperm to the treatment seawater, prior to the start of the experiment. The change in pH, i.e., deviation from starting value, increased with increasing sperm concentration (Fig. 3) with a near 0.6 unit pH change in the most concentrated sperm solutions (10^7^ sperm ml^−1^). pH continued to decline during the subsequent ∼180 minutes of the experiment, however, these changes were small relative to those that occurred prior to the experiment (Table S2). Despite the rapid changes in pH at higher sperm concentrations (10^6^ and 10^7^ sperm ml^−1^), high levels of fertilization (≥95%) were achieved (Table 2).

## Discussion

By fitting fertilization curves for each temperature*CO_2_ treatment, we show that increases in temperature (+3°C) and *p*CO_2_ (+400 µatm), within the ranges projected for this century, negatively impact fertilization success of *A. tenuis*. Maximum fertilization (Fert_max_) did not differ among treatments, but the fertilization curve shifted horizontally such that the sperm concentration required to achieve half of the maximum fertilization (EC50) increased with the addition of experimental factors. These results indicate that sperm concentrations that yield high fertilization success under present-day conditions are likely to be limiting as the oceans continue to warm and acidify. The nature of the effect, a shift in the fertilization curve to higher sperm concentrations without a reduction in %Fert_max_, suggests that both temperature and CO_2_ influence fertilization through effect(s) on sperm viability or sperm-egg interactions [12], [15]. Decreased sperm motility in response to CO_2_-induced acidification was recently demonstrated in *Acropora digitifera* [19], [20] and may provide a mechanistic basis for the results observed here.

The effects of CO_2_ and temperature on fertilization were dependent on the sperm concentration. Maximum differences between treatments were observed within the range of 10^2^–10^4^ sperm ml^−1^, whereas all treatments achieved ≥95% fertilization success at sperm concentrations of 10^5^–10^7^ sperm ml^−1^. Moreover, despite rapid changes in pH at higher sperm concentrations (10^6^ and 10^7^ sperm ml^−1^), high levels of fertilization (≥95%) were achieved, further demonstrating that while acidification inhibits fertilization success at non-saturating sperm concentrations, optimal sperm concentrations are capable of resulting in high fertilization success. These results are consistent with ecotoxicological studies on other marine invertebrates [12], [15] and support the concept that the effect of an environmental toxicant on fertilization success is dependent on the sperm concentration, with lower, non-saturating sperm concentrations typically showing the highest sensitivity. This is thought to occur because the addition of a toxicant typically acts to reduce the number of sperm-egg interactions, either by killing sperm or reducing sperm-egg contact efficiency [15]. Importantly, at ‘optimal’ sperm concentrations, enough interactions occur that this effect is not detected. Unfortunately, many recent studies have evaluated the effect of environmental stressors on fertilization using a single sperm concentration that has previously been identified as optimal, severely limiting our ability to make useful predictions about the likely impact of these stressors on sexual reproduction [13]. The dependence of treatment effects on the sperm concentration suggests that the susceptibility of fertilization success to warming and acidification may vary among coral populations; populations that are likely to be sperm-limited (e.g., low density populations and/or populations that experience high wind/wave activity) may be more likely to experience declines in fertilization, while those that currently experience optimal concentrations may be more resistant.

Currently, robust estimates of *in situ* sperm concentrations and fertilization rates are lacking for corals. Available information indicates that while optimal sperm concentrations are obtainable for some species under certain conditions [21], sperm concentrations and fertilization rates are highly variable in both space and time [14], [21] and are generally assumed to be limiting for most sessile, broadcast-spawning marine invertebrates [22]. Because sperm concentration depends on a number of factors including population density, time after spawning, and wind and water turbulence, identifying concentrations that are ecologically relevant is complex. Despite these challenges, there is an urgent need to characterize naturally-occurring fertilization conditions if we are to better gauge the threat that global warming and ocean acidification pose to sexual reproduction of corals.

The effects of CO_2_ and temperature on fertilization of *A. tenuis* are similar to the independent effects of these parameters observed in other studies [18], [23]. Albright et al. [18] showed that fertilization of a Caribbean congener, *Acropora palmata*, was negatively affected by elevated *p*CO_2_ (673 and 998 µatm); similar to this study, the effect of *p*CO_2_ on fertilization success was dependent on the sperm concentration and was exacerbated at lower, non-saturating concentrations. *Acropora millepora* experienced reduced fertilization and a higher frequency of embryonic abnormalities at 32°C (+4°C), and fertilization ceased altogether at 34°C (+6°C) [23]. However, in the same study, three other coral species, *Favites abdita*, *Favites chinensis* and *Mycedium elephantotus* were fertilized and developed normally at temperatures up to 32°C (+5°C), indicating that the susceptibility to climate change may differ among coral taxa. More research is needed to determine the generality of the response observed in this study; multi-stressor experiments should be emphasized as species may respond differently to dual stressors. Identifying species-specific differences in the susceptibility of fertilization and recruitment to global warming and ocean acidification may implicate future changes in community structure.

While only a handful of studies have investigated the effects of *p*CO_2_ and temperature on coral fertilization, the effects of these factors on fertilization of other spawning marine invertebrates has been the subject of many recent investigations, though results are equivocal [9], [13], [24]. For example, Havenhand et al. [25] reported acidification-induced reductions in fertilization for the sea urchin *Heliocidaris erythrogramma*; however, Byrne et al. [26], [27], [28] observed no effect in the same species. Similarly, Havenhand et al. [29] and Parker et al. [30] report contrasting results for fertilization success of the bivalve species, *Crassostrea gigas*, exposed to acidified seawater. It is not yet clear whether discrepancies between studies stem from species-specific differences or variations in the methodologies employed (reviewed in [24]). Consequently, there is an increasing awareness of the need for standardized methodologies to facilitate comparisons between studies.

Interpretation of laboratory results is further confounded by our inability to accurately replicate conditions in an organism's natural environment. For example, scintillation vials limit water movement, which may affect fertilization dynamics (although the use of multiple sperm concentrations partially accounts for diffusive effects of the open ocean); also, experimental set-up time necessitates a delay in gamete contact, which may or may not occur in nature. This time component, and the associated gamete aging, may affect the susceptibility of gametes to experimental stress. Despite these limitations, results of fertilization experiments provide valuable insight regarding the potential consequences of a warming and acidifying ocean on sexual reproduction of spawning marine organisms.

Recent investigations indicate that elevated temperatures and *p*CO_2_ levels also impact coral reproduction and recruitment through effects on post-fertilization processes (e.g., larval development, survival, settlement, metamorphosis, and early post-settlement growth). Temperatures exceeding 30°C have negative effects on the development and survival of some coral species [31], [32], [33]. Effects of temperature on larval settlement are more complex, as thermal stress has been shown to both increase [34] and decrease [34], [35] settlement. Elevated *p*CO_2_ does not appear to affect embryonic development [36], but larval respiration [37], [38] may be affected. *p*CO_2_ also affects larval settlement, though it appears to do so indirectly through impacts on the epilithic algal community [38], [39]; under future ocean acidification scenarios, certain species of crustose coralline algae (e.g., *Titanoderma prototypum*), that promote larval metamorphosis and facilitate survival [11] appear to lose out to less desirable taxa [39]. Acidification also reduces post-settlement growth and calcification [13], [18], [40], [41] impairing the ability of new recruits to effectively compete for benthic area. Given the range of life history stages and processes that appear negatively impacted by global warming and ocean acidification, carryover effects, and/or compounding effects on successive life history stages are likely.

What has been and will be the cumulative toll of climate change and ocean acidification on coral recruitment? Many of the dominant framework-building corals in the Caribbean (broadcast-spawning species – *Acropora palmata, A. cervicornis, Montastrea spp*.) have been observed to suffer from reproductive and/or recruitment failure [42]. Lack of historical baselines limits our understanding of whether recruitment of these species declined or has always been low. Furthermore, it remains unknown if, or to what extent, changing temperatures and CO_2_ levels have contributed to this failure, though it has been speculated that they are already impacting juvenile coral growth in the Caribbean [43]. As coral populations decline worldwide, successful reproduction and recruitment will become more challenging. The results of this study suggest that, irrespective of changes in current population structure, sexual reproduction of corals will be compromised as warming and acidification narrow the window for successful fertilization. Future work should investigate the presence of species-specific adaptations that may infer resistance to declines in sexual reproduction. Developing strategies to manage for reproductive and recruitment success may prove a necessary step towards promoting the sustainability, persistence, and recovery of coral populations.

## Supporting Information

Table S1 **Tukey's Multiple Comparisons of LogEC50 values by treatment.**
(DOCX)

Table S2 **Respiratory alterations of target pH values by treatment and sperm concentration (N = 2).**
(DOCX)
