# Supplementary material for: Projected Near-Future Levels of Temperature and pCO2 Reduce Coral Fertilization Success
Source: PLoS One. 2013 Feb 14;8(2):e56468. doi: 10.1371/journal.pone.0056468 (PMC3572969; doi:10.1371/journal.pone.0056468)
Supplement: Table S1 — Tukey's Multiple Comparisons of LogEC50 values by treatment. (DOCX) [file pone.0056468.s001.docx]

**Table S1.** Tukey’s Multiple Comparisons of LogEC50 values by treatment.

| Treatment Comparison | Mean Difference | q | P < 0.05 |
| --- | --- | --- | --- |
| 400 µatm x 27⁰C v 800 µatm x 27⁰C | -0.9140 | 16.27 | Y |
| 400 µatm x 27⁰C v 400 µatm x 30⁰C | -0.8020 | 14.28 | Y |
| 400 µatm x 27⁰C v 800 µatm x 30⁰C | -1.633 | 29.07 | Y |
| 800 µatm x 27⁰C v 400 µatm x 30⁰C | 0.1120 | 1.994 | N |
| 800 µatm x 27⁰C v 800 µatm x 30⁰C | -0.7190 | 12.80 | Y |
| 400 µatm x 30⁰C v 800 µatm x 30⁰C | -0.8310 | 14.79 | Y |
