# Supplementary material for: Projected Near-Future Levels of Temperature and pCO2 Reduce Coral Fertilization Success
Source: PLoS One. 2013 Feb 14;8(2):e56468. doi: 10.1371/journal.pone.0056468 (PMC3572969; doi:10.1371/journal.pone.0056468)
Supplement: Table S2 — Respiratory alterations of target pH values by treatment and sperm concentration (N = 2). (DOCX) [file pone.0056468.s002.docx]

**Table S2.** Respiratory alterations of target pH values by treatment and sperm concentration (N=2).

|  | **SW Control** | **10^3^**  **sperm ml^-1^** | **10^4^**  **sperm ml^-1^** | **10^5^**  **sperm ml^-1^** | **10^6^**  **sperm ml^-1^** | **10^7^**  **sperm ml^-1^** |
| --- | --- | --- | --- | --- | --- | --- |
| **400 µatm x 27⁰C** |  |  |  |  |  |  |
| **pH Initial: 8.074 ± 0.004** |  |  |  |  |  |  |
| **pH Start Experiment** | **8.074 ± 0.004** | **8.067 ± 0.001** | **8.068 ± 0.005** | **8.050 ± 0.002** | **7.865 ± 0.002** | **7.525 ± 0.004** |
| **pH End Experiment** | **8.078 ± 0.002** | **8.071 ± 0.004** | **8.05 ± 0.01** | **8.03 ± 0.03** | **7.752 ± 0.006** | **7.444 ± 0.002** |
|  |  |  |  |  |  |  |
| **800 µatm x 27⁰C** |  |  |  |  |  |  |
| **pH Initial: 7.860 ± 0.007** |  |  |  |  |  |  |
| **pH Start Experiment** | **7.860 ± 0.007** | **7.803 ± 0.000** | **7.816 ± 0.001** | **7.796 ± 0.002** | **7.56 ± 0.01** | **7.30 ± 0.01** |
| **pH End Experiment** | **7.878 ± 0.006** | **7.807 ± 0.001** | **7.84 ± 0.01** | **7.795 ± 0.007** | **7.47 ± 0.02** | **7.277 ± 0.004** |
|  |  |  |  |  |  |  |
| **400 µatm x 30⁰C** |  |  |  |  |  |  |
| **pH Initial: 7.991 ± 0.002** |  |  |  |  |  |  |
| **pH Start Experiment** | **7.991 ± 0.002** | **7.99 ± 0.00** | **7.991 ± 0.002** | **7.970 ± 0.002** | **7.761 ± 0.004** | **7.435 ± 0.004** |
| **pH End Experiment** | **8.004 ± 0.008** | **8.009 ± 0.001** | **7.995 ± 0.002** | **7.972 ± 0.002** | **7.646 ± 0.009** | **7.34 ± 0.01** |
|  |  |  |  |  |  |  |
| **800 µatm x 30⁰C** |  |  |  |  |  |  |
| **pH Initial: 7.794 ± 0.002** |  |  |  |  |  |  |
| **pH Start Experiment** | **7.794 ± 0.002** | **7.701 ± 0.008** | **7.741 ± 0.001** | **7.720 ± 0.001** | **7.459 ± 0.006** | **7.202 ± 0.007** |
| **pH End Experiment** | **7.823 ± 0.001** | **7.743 ± 0.008** | **7.782 ± 0.009** | **7.75 ± 0.00** | **7.374 ± 0.001** | **7.19 ± 0.02** |

^a^ pH Initial is the seawater pH following bubbling with air or CO_2_ but prior to the addition of sperm; pH Start Experiment is the seawater pH at the start of the fertilization experiment, approximately 90 minutes after the addition of sperm; pH End Experiment is the seawater pH at the end of the fertilization experiment, approximately 270 minutes after the addition of sperm. Changes were not measured in 10^2^ sperm ml^-1^ as preliminary work showed that changes in this concentration were comparable to seawater controls.
